# Retraction: Synergistic Apoptosis of CML cells by buthionine sulfoximine and hydroxychavicol correlates with activation of AIF and GSH-ROS-JNK-ERK-iNOS pathway

**DOI:** 10.1371/journal.pone.0319529

**Published:** 2025-02-24

**Authors:** The PLOS One Editors

After this article [1] was published, concerns were raised regarding results presented in Figs 2, 4, 6, and 7.

Specifically:

- Regions of the following FACS panels appear more similar than would be expected from independent results:
  - Fig 2A Veh. Cont., BSO (36h), and HCH (36h) panels
  - Fig 2C Veh. Cont. panel and BSO (24h) panel
  - Fig 4E Veh. Cont., GME (2mM), and GME+BSO+HCH dot plot panels
  - Fig 7D Veh. Cont. Cont. siRNA panel and Veh. Cont. eNOS siRNA panel
- In Fig 6A pJNK blot, when levels are adjusted to visualize background, lanes 1-2 appear similar to lanes 3-4
- In Fig 6C, the Veh. Cont. Cont. siRNA panel appears similar to Veh. Cont. JNK1 siRNA panel
- The following FACS histograms appear similar:
  - Fig 6G None Cont siRNA, Fig 7B Veh. Cont Cont. siRNA, Fig 7B Veh. Cont iNOS siRNA, and Fig 7B Veh. Cont nNOS siRNA panels

The corresponding author disagrees with the above concerns raised with the FACS panels presented in Figs 2, 4, 6, and 7, stating that in flow cytometry it is possible that some regions appear identical in datasets representing different experimental conditions. The corresponding author did not provide the raw FACS data files for editorial review. PLOS remains concerned that the results presented in these panels appear more similar than would be expected from independent samples.

The corresponding author states that errors were made during the preparation of the Fig 6C Veh. Cont. Cont. siRNA and Veh. Cont JNK1 siRNA panels, and the Fig 7B Veh. Cont Cont. siRNA and Veh. Cont iNOS siRNA panels. The corresponding author provided replacement panels from repeat experiments, but in the absence of the original data underlying the published results, these concerns cannot be fully resolved.

Regarding the similarities between the Fig 6G None Cont. siRNA panel and the Fig 7B Veh. Cont Cont. siRNA panel, the corresponding author states that the histograms are similar as the same treatment condition was tested twice on the same day.

The corresponding author states the original data underlying the published figures are no longer available due to the age of the article. The corresponding author provided replacement panels from repeat experiments to support Figs 2A, 2C, 6C, and 7B, but these were insufficient to resolve the concerns pertaining to the integrity of the published figures.

In light of the above unresolved concerns that question the integrity and reliability of the reported results and conclusions, the *PLOS One* Editors retract this article.

SB, AM, and NB did not agree with the retraction and stand by the article’s findings. AAC did not agree with the retraction. SKM responded but expressed neither agreement nor disagreement with the editorial decision. JC, SC, UC, and PJ either did not respond directly or could not be reached.
